# Jieduan–Niwan Formula Ameliorates Oxidative Stress and Apoptosis in Acute-on-Chronic Liver Failure by Suppressing HMGB1/TLR-4/NF-*κ*B Signaling Pathway: A Study In Vivo and In Vitro

**DOI:** 10.1155/2022/1833921

**Published:** 2022-07-15

**Authors:** Peng Fang, Bo Dou, Weixin Hou, Xiaoyi Wei, Jiajun Liang, Chongyang Ma, Qiuyun Zhang

**Affiliations:** ^1^Department of Infectious Diseases, The First Affiliated Hospital of Zhejiang Chinese Medical University (Zhejiang Provincial Hospital of Traditional Chinese Medicine), Hangzhou 310006, Zhejiang Province, China; ^2^Beijing Key Lab of TCM Collateral Disease Theory Research, School of Traditional Chinese Medicine, Capital Medical University, Beijing 100069, China

## Abstract

Jieduan-Niwan (JDNW) formula is a traditional Chinese medicine compound created by the famous Chinese medicine expert Professor Qian Ying, and has been used clinically for decades to treat acute-on-chronic liver failure (ACLF) and exhibits remarkable efficacy. However, the exact mechanism remains to be discovered. As an important hepatocyte damage-associated molecular patterns (DAMP) factor, high mobility group box 1 (HMGB1) is a potential therapeutic target as an accelerator of ACLF in the pathogenesis. Therefore, the present study investigated whether JDNW inhibits the overexpression and cytoplasmic translocation of HMGB1 in ACLF liver tissue and alleviates its mediated oxidative stress and apoptosis. In vivo, an immune-induced ACLF rat model was established, and then treated with JDNW for 5, 10, and 15 d. The results showed that a large number of cytoplasmic translocations of HMGB1 occurred in the ACLF group. And there was an increase in the expression of HMGB1 in the M-5 d group. After the intervention of JDNW, the overexpression and translocation of HMGB1 were inhibited. In vitro, D-GaLN caused an increase in the expression and translocation of HMGB1 in L02 cells. Similar to the inhibitor of HMGB1, JDNW serum alleviated this kind of increase. Further tests showed that JDNW attenuated ACLF-related oxidative stress and apoptosis, and the inhibition was associated with the regulation of TLR-4/NF-*κ*B signaling pathway. In conclusion, our present findings suggest that the therapeutic effect of JDNW on ACLF was associated with the inhibition of high expression and cytoplasmic translocation of HMGB1 during the acute injury phase, thus, attenuating oxidative stress injury and apoptosis induced by HMGB1/TLR-4/NF-*κ*B pathway.

## 1. Introduction

Acute-on-chronic liver failure (ACLF) is a life-threatening liver failure syndrome [1].

The main causes of ACLF vary widely between East and West, with hepatitis virus and alcohol abuse, respectively [2]. The pathogenesis of ACLF is very complicated, characterized by immune system disturbances and excessive inflammatory responses, which lead to the release of damage-associated molecular patterns (DAMPs) [3]. By binding to specific receptors, the inflammatory response is enhanced by DAMP and further recognized by the immune system; thereby releasing pro-inflammatory factors, triggering and aggravating the inflammatory response of the liver, which can eventually lead to liver failure [4, 5].

High mobility group box 1 (HMGB1) is a ubiquitous eukaryotic nuclear DNA-binding protein with diverse functions in and out of cells [6]. When infection or sterile injury occurs, HMGB1 translocates extracellularly, either actively by macrophages or by passive pathways in damaged cells, thereby becoming a DAMPs factor that binds to atopic receptors. It acts as a damage announcer, transmitting damage signals to adjacent cells [7]. Likewise, HMGB1 is a hepatocyte DAMP that modulates cell death patterns [8]. HMGB1 in peripheral blood of ACLF patients was much more than that of chronic hepatitis patients, which may be a potential target for improving the prognosis of ACLF [9]. In addition to its pro-inflammatory role, which has been extensively studied, recent studies have demonstrated that HMGB1 also plays an important role in mediating oxidative stress and apoptosis [10]. As a prooxidant, NF-*κ*B is induced and activated by recombinant HMGB1 through TLR-4-dependent NADPH oxidase to generate a large amount of intracellular reactive oxygen species [11]. In addition, HMGB1 is also related to apoptosis. By binding to DNA, HMGB1 is released in late apoptotic cells [12]. In hepatic ischemia-reperfusion injury, the released HMGB1 activates caspase-3-dependent apoptosis through the TLR-4 pathway [13].

Clinical treatment of ACLF is still limited, and liver transplantation is the only established treatment, but it is only suitable for a small number of patients (∼5%) due to scarcity of donor organs, cost, and contraindications. Traditional Chinese medicine (TCM) has irreplaceable advantages in the treatment of ACLF and is an important complementary and alternative therapy at present. Qian Ying, a master of traditional Chinese medicine, based on his lifelong learning and medical experience, created the Jieduan-Niwan (JDNW) formula to treat ACLF, which has a unique curative effect. JDNW was significantly better than the general treatment group in improving liver function and reducing mortality in ACLF patients [14]. JDNW is designed for the ACLF's pathogenesis of TCM “poison damage to the liver” and “loss of righteous qi.” In the formula, the role of bitter *Phyllanthus vulgaris*, Trichosanthis, Lysimachia, and Turmeric are used for “attacking and replenishing, detoxification, removing blood stasis and dampness, and strengthening righteousness turmeric.” *Salvia miltiorrhiza* are used for “clearing away heat and detoxification,” “removing phlegm and dampness,” “promoting gallbladder and reducing jaundice,” and “cooling blood and removing blood stasis.” Therefore, they share the responsibility of “Jieduan,” that is, preventing the disease from worsening. *Astragalus* and *Panax notoginseng* are used for “invigorating the spleen and qi” and “promoting blood circulation and regulating the liver.” Mistletoe, dried rehmannia root, and aconite “nourishes the yin of the liver and kidney, and the yang of the spleen and kidney.” Thus, it played the role of “Niwan,” which means to replenish the righteousness and exorcise evil [15]. Due to the good clinical effect of JDNW, the ACLF treatment guidelines of Integrative Chinese and Western Medicine propose that JDNW is the key method to treat ACLF [16]. However, the underlying scientific reasons for JDNW to alleviate ACLF have not been well studied, limiting our generalization and application of it. Several of our previous experiments showed that JDNW can reduce 24-hour mortality, reduce liver inflammation, and excessive apoptosis in ACLF model rats [17–19]. However, whether the specific mechanism of JDNW's treatment of ACLF is related to HMGB1, the important hepatocyte DAMP molecule remains unknown and thus warrants further research.

## 2. Materials and Methods

### 2.1. Animals

Specific pathogen-free male Wistar rats were purchased from Vital River Laboratory Animal Technology Co. Ltd. All the rats were housed in the Department of Laboratory Animals, Capital Medical University. They were reared under environmental conditions of temperature (25 ± 3°C) and humidity (60 ± 10%) with a 12-hour light-dark cycle. The animal feeding and handling were carried out in accordance with the “Guidelines for the Care and Use of Laboratory Animals.” The animal experiment ethics were approved by the Animal Care and Use Committee of Capital Medical University (No. AEEI-2019-067).

### 2.2. Preparation of JDNW

The formulation ingredients and herbal doses of each dose of JDNW are listed in Supplementary Table S1. The preparation of JDNW was as previously described. Briefly, soak all the herbs in distilled water for 4 hours. After boiling the aconitum with 1 L of distilled water for 30 minutes, add the rest of the herbs and boil for another 45 minutes to obtain the concoction of the first decoction. The herbs were then boiled again in 1 L of distilled water for 30 minutes to collect the second decoction. After mixing the two decoctions evenly, the preparation of JDNW was completed. Based on the results of our previous study, the optimal dose of JDNW formulation per rat was 21.7 g/kg/d. After concentrating the decoction to 4.34 g/mL, it was refrigerated at 4°C for intragastric administration of rats [17].

The protocol for the identification and qualification of the main chemical components of it were as previously described. Briefly, we established a fingerprint using HPLC-MS analysis to characterize the compounds of the JDNW formula and control the quality. Seven kinds of JDNW compounds, including catalpol, gallic acid, 3,4-dihydroxybenzaldehyde, chlorogenic acid, notoginsenoside R1, salvianolic acid B, and ginsenoside Rb1 were characterized and quantified [19].

### 2.3. Animal Modeling and Drug Administration

A total of 90 rats were randomly divided into the normal control group (*n* = 6) and the treatment group (*n* = 82). Immune liver injury induced by injection of human serum albumin (HSA) in the treatment group. As a xenogeneic serum, HSA elicits an immune response in rats, and the formed immune complex deposits stimulate collagen proliferation and cause liver fibrosis. The procedure consists of two steps: subcutaneous injection for sensitization (one injection on 0, 13, 23, and 33 d, respectively, each injection of HSA 4 mg) and tail vein injection to induce immune fibrosis (twice a week for a total of 6 weeks, the dose of HSA was gradually increased by 2.5 mg ⟶ 3 mg ⟶ 3.5 mg ⟶ 4 mg ⟶ 4.5 mg, and then maintained at 4.5 mg). After 6 weeks, the survived 74 rats in the treatment group were intraperitoneally injected with 400 mg/kg D-GaLN and 100 *μ*g/kg LPS for acute liver injury to establish an ACLF model, as described previously [19]. After the ACLF model was established, the survived 72 rats were randomly divided into the JDNW group and Model (M) group. The above two groups were randomly divided into three subgroups, namely the JDNW/M group (5, 10, and 15 d; *n* = 12 per group). JDNW treatment started 24 hours after the acute injury, and the treatment group was given intragastric administration for 5, 10, and 15 d. The NC group and the M group were continuously given a 0.9% equal dose of normal saline. Samples for research were collected from rats in each group at 5, 10, and 15 d after treatment for analysis. The non-JDNW intervention groups were given the same amount of normal saline by gavage. After anesthesia with 1% sodium pentobarbital (40 mg/kg), blood was drawn from the abdominal aorta, placed at room temperature for 30 minutes, and centrifuged (3000 rpm, 15 minutes) to separate and collect serum. Liver tissue was rapidly collected and frozen in liquid nitrogen. The euthanasia method for rats was performed by cervical dislocation. The obtained specimens were stored at −80°C.

### 2.4. Determination of Serum Alanine Aminotransferase (ALT), Aspartate Aminotransferase (AST), Total Bilirubin (TBiL), and Prothrombin Times (PTs)

Serum and plasma were collected in non-anticoagulated and anticoagulated tubes, and centrifuged for 15 minutes at 3000 rpm (Sigma, USA). Levels of ALT, AST, TBiL, and PT were detected by assay kits. All steps are followed as per the manufacturer's instructions.

### 2.5. Histological Evaluation

After fixation, paraffin-embedded, sectioned 5 *μ*m thick, stained with hematoxylin and eosin (H and E), and the pathological pictures were acquired with a panoramic scanner (Leica Aperio AT2) for pathological sections.

### 2.6. JDNW Serum Preparation

To further explore the pharmacodynamic mechanism of JDNW, we prepared JDNW serum for in vitro experiments. Briefly, rats were given an equal amount of distilled water and 21.7 g JDNW crude drug/kg/day by gavage for 7 d to obtain control serum and JDNW serum, respectively. After anesthetizing the rats with a small animal anesthesia machine (Matrx VMR, Midmark), the abdominal aortic blood collection method was used for sterile collection. After standing vertically at 4°C for 4 hours, the blood sample was centrifuged at 3000 rpm/min for 30 minutes at 4°C, and then inactivated at 56°C for 30 minutes. Filtered by a microfiltration membrane (0.22 um), the samples were stored at −80°C.

### 2.7. MDA and GSH Content in ACLF Rats

MDA and GSH in liver tissues were detected with assay kits, respectively (Beyotime, China; cat: S0131; Beyotime, China; cat: S0053). Measure the content of MDA and GSH with a microplate reader at the absorption wavelengths of 532 and 412 nm, respectively. The contents of MDA and GSH are expressed as nmol/mg protein.

### 2.8. Cell Culture

Normal human hepatocytes (L02 cells) were used for in vitro experiments. L02 cells were obtained from the Cell Bank of Type Culture Collection of the Chinese Academy of Sciences (Shanghai, China) and were maintained in DMEM media supplemented with 10% (v/v) fetal bovine serum (FBS), 37°C, 5% CO_2_. L02 cells in the logarithmic growth phase were digested with 0.25% trypsin to prepare cell suspension. The cells were inoculated with 3 × 10^6^ cells in 6-well plates and incubated in an incubator for 12 hours. The L02 cells were randomly divided into the NC group, D-GaLN group, JDNW group, and HMGB1 inhibitor (glycyrrhizin, GLY) group. Cultivating intervention conditions: NC group and D-GaLN group were in the medium containing 10% control serum, GLY group was treated with 10% control serum medium (containing 1 mmol/L GLY), and JDNW group was treated with JDNW serum at different concentrations (2.5%, 5%, and 10%), the total serum concentration was supplemented to 10% with the control serum. The intervening concentration of D-GaLN was screened by cell viability assay.

### 2.9. CCK-8

The Cell Counting Kit-8 (CCK8) assay (Beijing, China; catalog: AQ308) was used to evaluate the effect of D-GaLN and JDNW serum on L02 cell viability. After treatment with D-GaLN and/or JDNW serum for the indicated times, cells from different groups were incubated with the CCK-8 reagent for 2 hours. Measure the optical density of each well by using a microplate reader (Thermo Scientific, USA).

### 2.10. Western Blot

Total protein was extracted from L02 cells and liver tissue samples by radioimmunoprecipitation assay lysis buffer (RIPA; Applygen, China; cat: C1053). The nuclear and cytoplasmic protein extraction kit (Beyotime, China; catalog: P0028) was used to separate cytoplasmic and nuclear proteins. Protein concentration was determined with bicinchoninic acid (BCA) protein reagent (Beyotime, China; catalog: P0012). The protein lysate was electrophoresed, transferred to the membrane, and blocked with nonfat milk powder. The primary antibody was incubated overnight at 4°C, and after washing with TBST, the secondary antibody was incubated for 1 hours. Finally, a chemiluminescence reagent (NCM Biotech, China; cat: P10100) was used to detect the reaction. An ImageQuantLAS4000 (GE Co., USA) chemiluminescence imaging system was used to acquire visual images of target proteins. Quantify protein expression using Image J software version 1.80, and expressed as a percentage of the control.

### 2.11. Immunostaining of 4-HNE and HMGB1

After deparaffinization and antigen retrieval, paraffin sections were incubated with bovine serum albumin (BSA) and blocked. For L02 cells, after exposure to the indicated experimental conditions, they were then fixed with 4% paraformaldehyde and blocked with 5% BSA. Pathological sections and cell slides were incubated with primary antibodies overnight at 4°C. After being washed with PBS, the slides were incubated with TIRTC-labeled secondary antibodies for 2 hours at 37°C in the dark. Anti-fluorescence decay sealing solution (contains DAPI) sealed the slides. Fluorescence images were acquired with a confocal microscope (Leica TCS SP8) and quantified with Image J software version 1.80.

### 2.12. Real-Time Quantitative Polymerase Chain Reaction (qRT-PCR) to Detect the Expression of HMGB1

Total mRNA from liver tissue was extracted with RNA prep pure Tissue Kit (TIANGEN Biotech, China; catalog: DP431) and reverse transcribed into cDNA. The qRT-PCR reaction system was established by SuperReal PreMix Plus Kit (Tiangen Biotechnology, China; catalog: FP215) with GAPDH as the internal control. The amplification procedure was as follows: predenaturation: 95°C, 5 minutes, 1 cycle; amplification: 95°C, 10 s, 60°C, 30 s, a total of 40 cycles. As final, statistical analysis of gene expression was performed by the 2^−ΔΔCT^. The specific primer sequences are in Supplementary Table S2.

### 2.13. Tunel

TdT-mediated dUTP Nick end labeling (TUNEL) detection and fluorescein in situ cell death assay kit (KeyGEN BioTECH, China; catalog: KGA7072) were used to detect the apoptotic response of L02 cells and liver tissues, according to the manufacturer's instructions.

### 2.14. Measurement of Reactive Oxygen Species (ROS)

ROS in L02 cells was measured by using a reactive oxygen species detection kit (Beyotime, China; cat: S0033). Seeded the cells in a 6-well plate, after being exposed to the specified experimental conditions, incubated with 10 mM DCFH-DA at 37°C for 30 minutes. The cells were then harvested by trypsinization and centrifugation, and the level of intracellular ROS was detected by flow cytometry.

### 2.15. Statistical Analysis

All the data were analyzed by Prism 8.0 and expressed as mean ± standard deviation (SD). Statistical comparison among multiple groups was performed using one-way ANOVA followed by Tukey's post hoc test (data are normally distributed and homogeneity of variance, results were expressed as mean ± standard deviation (SD)) or Kruskal–Wallis test followed by Dunn's test (data are non-normally distributed or without homogeneity of variance, results were expressed as the median (min − max)). *p* < 0.05 is considered statistically significant.

## 3. Results

### 3.1. Effects of JDNW on Liver Function and Pathology in ACLF Rats

**Figure 1:**
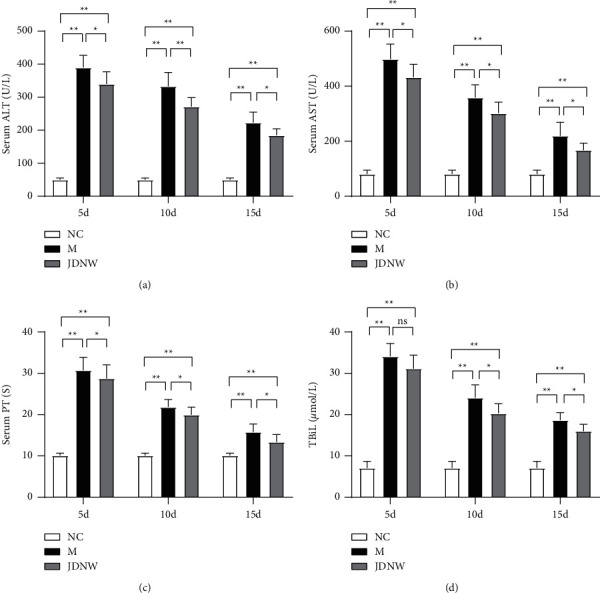
Effects of Jieduan-Niwan (JDNW) formula intervention on liver function in acute-on-chronic liver failure (ACLF) rats at different time points. (a) The serum levels of alanine aminotransferase (ALT); (b) the serum levels of aspartate aminotransferase; (c) the serum levels of total bilirubin (TBiL); (d) prothrombin times (PTs). Each bar represents the mean ± SD of *n* = 5–6 (^*∗*^*p* < 0.05, ^*∗∗*^*p* < 0.01, “ns” *p* > 0.05).

**Figure 2:**
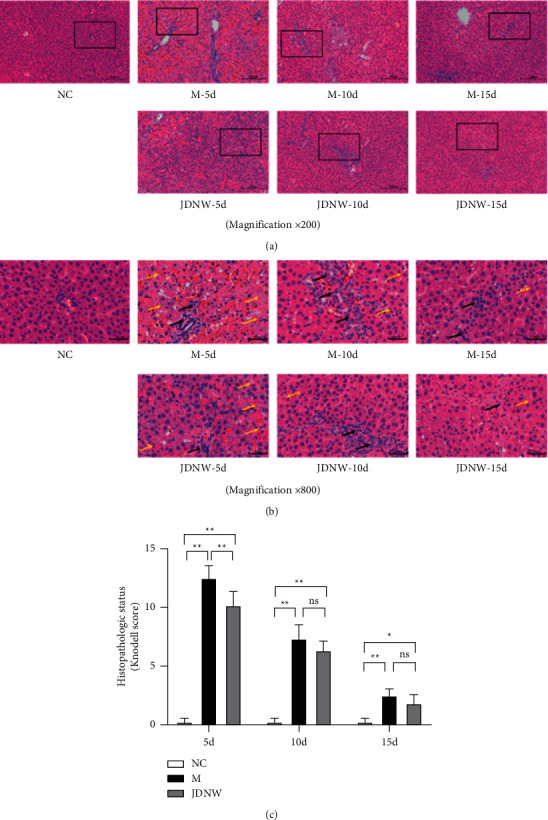
Effects of JDNW intervention on liver histopathology in ACLF rats at different time points. (a, b) Hematoxylin and eosin (H&E) staining to evaluate the pathological changes in rat's liver. Magnification 200x, 800x; scale bar: 200 *μ*m, 50 *μ*m. Infiltration of inflammatory cells (Black arrows), necrotic hepatocytes (Yellow arrows). (c) Evaluation of histopathological status (Knodell score). Each bar represents the mean ± SD of *n* = 5–6, (^*∗*^*p* < 0.05, ^*∗∗*^*p* < 0.01, “ns” *p* > 0.05).

As shown in Figure 1, serum levels of ALT, AST, TBiL, and PT were determined to evaluate the effect of JDNW on liver function. Compared with the model group, at the same time point, the liver function of the JDNW treatment group was significantly improved at 5, 10, and 15 d. Pathologically, liver cells in the ACLF group were arranged disorderly, inflammatory cells were infiltrated, hepatic sinusoids were dilated and hemorrhaged, and a large number of necrotic liver cells were present. Next, we performed a histopathological status assessment (Knodell score) to assess the level of inflammation and necrosis in liver tissue. Compared with the model group, the treatment of JDNW statistically reduced the Knodell score of liver tissue in the 5 d group (Figure 2).

### 3.2. Effects of JDNW on Oxidative Stress

**Figure 3:**
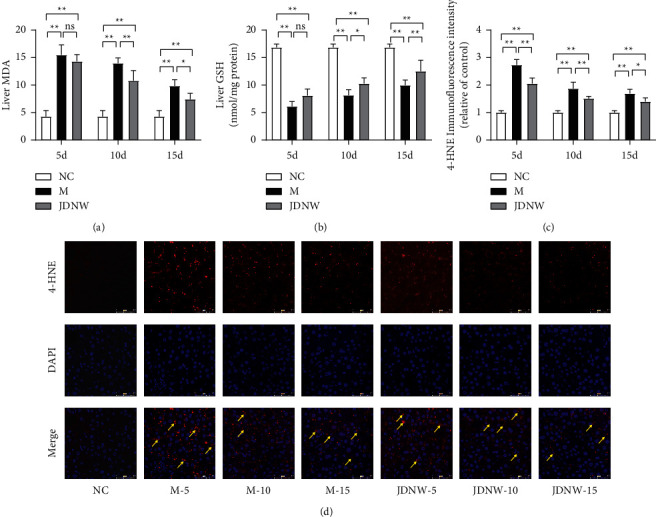
Effects of JDNW on oxidative stress injury of liver tissue in ACLF rats. (a) Malondialdehyde (MDA) content; (b) glutathione (GSH); (c, d) immunofluorescence results of 4-hydroxynonenal (4-HNE). Magnification 400x scale bar: 50 *μ*m. Each bar represents the mean ± SD of *n* = 5–6 (^*∗*^*p* < 0.05, ^*∗∗*^*p* < 0.01, “ns” *p* > 0.05).

As shown in Figure 3, the content of MDA and GSH, as well as the accumulation level of 4-HNE were examined to evaluate the effect of JDNW on oxidative stress. Compared with the NC group, 4-HNE had more accumulation in ACLF model liver tissue. Likewise, the MDA contents of the ACLF group increased, while the contents of GSH decreased, which was obvious in the M-5 d group. After JDNW treatment, the levels of 4-HNE and MDA in liver tissue decreased, and the level of GSH increased, indicating that the intervention of JDNW significantly reduced the oxidative stress damage in ACLF liver tissue.

**Figure 4:**
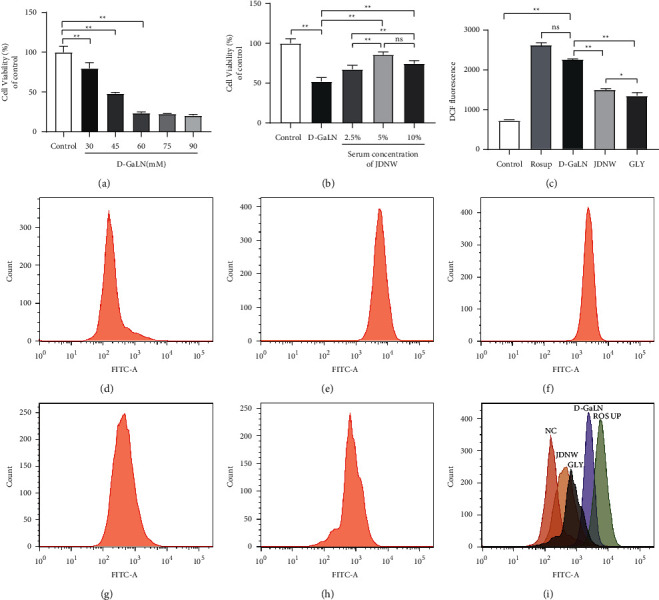
Effects of JDNW on D-galactosamine (D-GaLN)-induced cell damage and reactive oxygen species production in L02 cells. (a, b). The Cell Counting Kit-8 (CCK8) test was used to analyze cell viability. (a) Cells were treated with different concentrations of D-GaLN (30, 45, 60, 70, and 90 mM) and (b) pretreated with different concentrations of JDNW serum (2.5%, 5%, and 10%) for 12 hours and then cotreated with D-GaLN (45 mM) for 11 hours. (C–I) Flow cytometric analysis of ROS levels in L02 cells. (c) ROS statistics, (d) control group, (e) Rosup group, (f) D-GaLN (45 mM) group, (g) cotreated with JDNW (5%) and D-GaLN (45 mM) group, (h) cotreated with GLY (1 mM) and D-GaLN (45 mM), (i) aggregate results. Each bar represents the mean ± SD of *n* = 3 (^*∗*^*p* < 0.05, ^*∗∗*^*p* < 0.01, “ns” *p* > 0.05).

Meanwhile, we conducted a CCK-8 test in vitro, and confirmed that JDNW serum can attenuate D-GaLN damage to L02 cells (Figures 4(a) and 4(b)). Next, to further observe the effect of JDNW serum on oxidative stress damage, we measured the fluorescence intensity of DCF by flow cytometry to analyze the accumulation of ROS in L02 cells in vitro. As shown in (Figures 4(c)–4(i)), D-GaLN significantly increased the accumulation of ROS in L02 cells, while the JDNW group was similar to glycyrrhizin (GLY; a specific HMGB1 inhibitor) [20, 21], significantly reduced the accumulation of ROS in L02 cells.

### 3.3. The Amelioration of Apoptosis by JDNW May be Related to Mitochondrial Pathway

**Figure 5:**
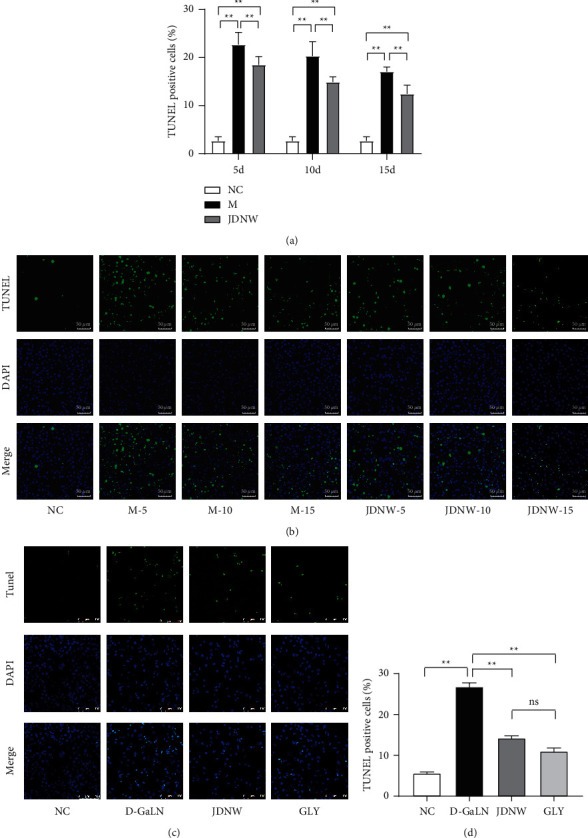
TdT-mediated dUTP Nick end labeling (TUNEL) staining to detect the effect of JDNW on the apoptosis rate of hepatocytes. (a, b) The effect of treatment at different time points on hepatocyte apoptosis in ACLF rats: 10 random fields/per slice, 3 random slices/per sample, representative of 5–6 rats/group. (c, d) Effects of JDNW serum on apoptosis of L02 cells induced by D-GaLN. Data are presented from three independent experiments. TUNEL-positive cells (green fluorescence). Each bar represents the mean ± SD (^*∗*^*p* < 0.05, ^*∗∗*^*p* < 0.01, “ns” *p* > 0.05).

Next, we performed TUNEL staining to detect the apoptosis of ACLF rat liver tissue and L02 cells. As shown in (Figures 5(a) and 5(b)), the apoptosis rate of hepatocytes (TUNEL-positive cell rate) of the ACLF group increased, and the M-5 d group was the highest. After JDNW treatment, the apoptosis rate of hepatocytes was significantly lower than that of the model group at the same time point. Likewise, in vitro, the intervention of JDNW serum significantly reduced the apoptosis of L02 cells caused by D-GaLN, and its effect was similar to that of GLY (Figures 5(c) and 5(d)).

**Figure 6:**
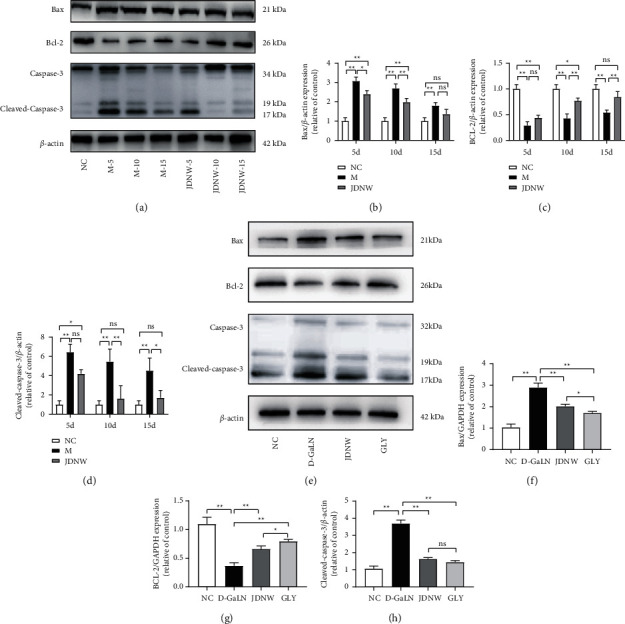
Effects of JDNW on the expression of Bax, Bcl-2, and Caspase-3 in vitro and in vivo. (a–d) Effects of JDNW treatment at different time points on mitochondrial apoptosis-related proteins in liver tissues of ACLF rats. (e–h). Effects of JDNW serum on mitochondrial apoptosis-related proteins of L02 cells. The data on quantified protein expressions were normalized by related *β*-actin. Each bar represents the mean ± SD of *n* = 3 (^*∗*^*p* < 0.05, ^*∗∗*^*p* < 0.01, “ns” *p* > 0.05).

Further, we examined the effect of JDNW on mitochondrial pathway apoptosis-related proteins. As shown in (Figures 6(a)–6(d)), in ACLF rats' liver tissue, the expression of cleaved-caspase-3 and Bax increased, and the anti-mitochondrial pathway apoptosis protein Bcl-2 decreased. While the treatment of JDNW reversed this kind of change compared with the model group during the same period. In vitro, the intervention of JDNW serum also reversed the increase of cleaved-caspase-3 and Bax, and the decrease in Bcl-2 caused by D-GaLN, similar to the effect of an inhibitor of HMGB1 (GLY; Figures 5(e)–5(h)).

### 3.4. Effects of JDNW on HMGB1

**Figure 7:**
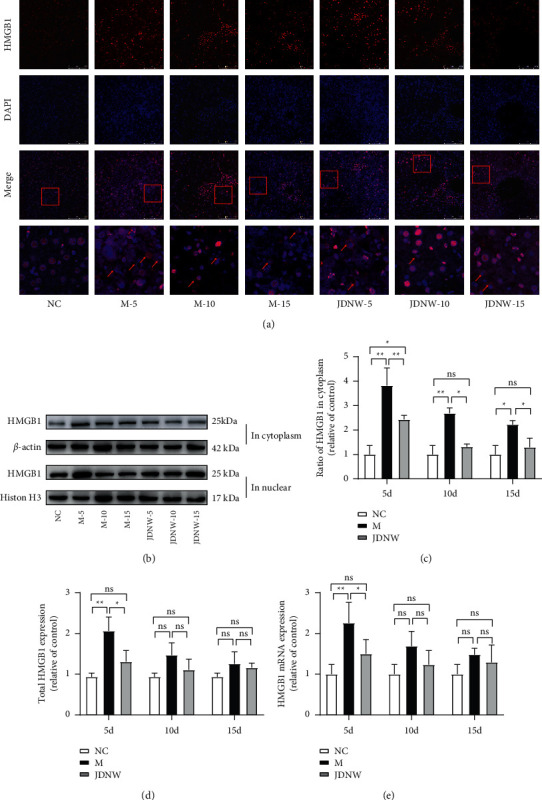
Effects of JDNW on the expression and translocation of HMGB1 in ACLF rats. (a) Immunofluorescence staining of HMGB1 at different time points. Scale bar: 100 *μ*m; HMGB1 in the cytoplasm (red arrows). (b) Western blot detection of HMGB1. (c) Ratio of HMGB1 in the cytoplasm and (d) total HMGB1 by western blot assay. (e) HMGB1 mRNA expression was detected by qRT-PCR. Each bar represents the mean ± SD of *n* = 3 (^*∗*^*p* < 0.05, ^*∗∗*^*p* < 0.01, “ns” *p* > 0.05).

**Figure 8:**
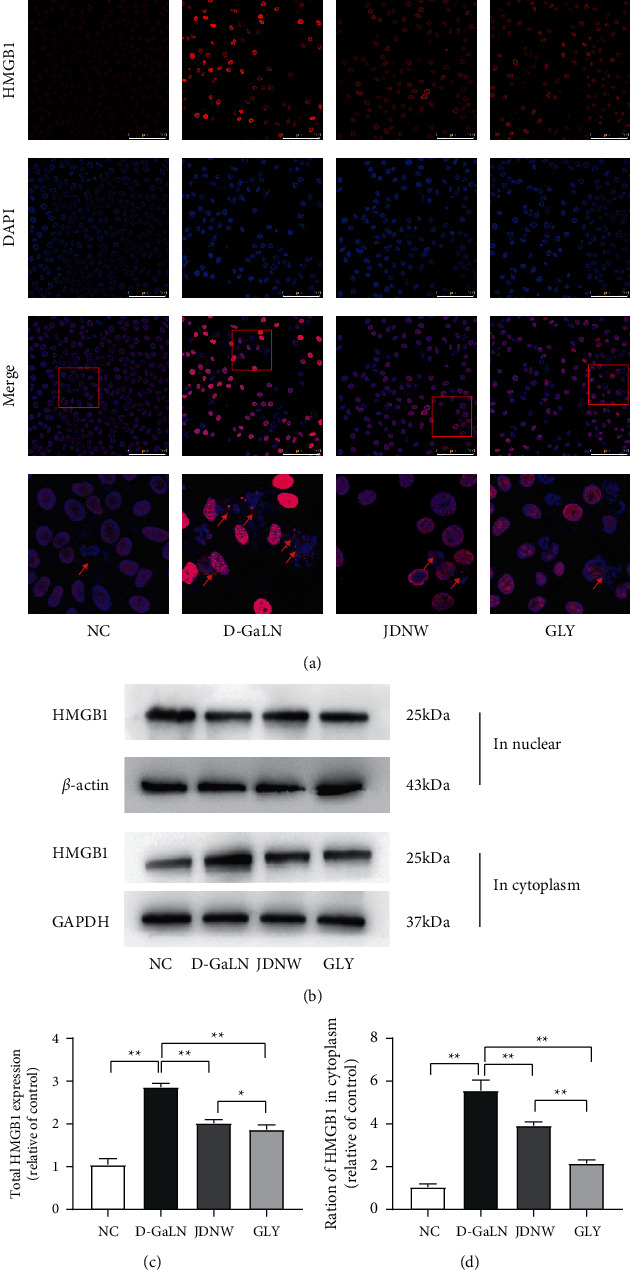
The effect of JDNW serum on the expression and translocation of HMGB1 in L02 cells. (a) Immunofluorescence staining of HMGB1. Scale bar: 100 *μ*m; HMGB1 in the cytoplasm (red arrows). (b) Western blot detection of HMGB1. (c) Total HMGB1 and (d) ratio of HMGB1 in the cytoplasm by western blot assay. Each bar represents the mean ± SD of *n* = 3 (^*∗*^*p* < 0.05, ^*∗∗*^*p* < 0.01, “ns” *p* > 0.05).

Our previous study showed the expression and translocation of HMGB1 were associated with oxidative stress and mitochondrial pathway apoptosis [22]. Therefore, we performed immunofluorescence, qRT-PCR, and western blotting to detect the expression and distribution of HMGB1, and the results (Figure 7) showed that the cytoplasmic distribution of HMGB1 in the ACLF group increased significantly. The total expression of HMGB1 in the M-5 d group was significantly increased, indicating that there was an overexpression of HMGB1 in the early period of ACLF injury. After JDNW treatment, the intracytoplasmic translocation and the overexpression of HMGB1 were significantly reduced. Also, the overexpression of HMGB1 in the early stage of ACLF injury was significantly reduced. In vitro, the intervention of D-GaLN resulted in the overexpression and the translocation of HMGB1 in L02 cells. However, after the intervention of JDNW serum and HMGB1 inhibitor-GLY, the increase was suppressed (Figure 8). These results indicated that JDNW inhibited the overexpression and intracytoplasmic translocation of HMGB1.

### 3.5. Effects of JDNW on HMGB1/TLR-4/NF-*κ*B Signaling Pathway

**Figure 9:**
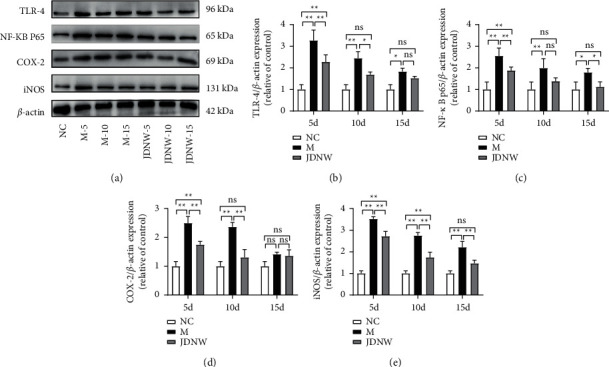
Effect of JDNW on HMGB1/TLR-4/NF-*κ*B pathway in ACLF rats. The protein expression levels in liver tissues of TLR-4, NF-*κ*B P65, iNOS, and COX-2 were evaluated by western blotting. (a) Representative immunoblots. (b–e) The relative protein expression results of TLR-4, NF-*κ*B P65, iNOS, and COX-2. Each bar represents the mean ± SD of *n* = 3 (^*∗*^*p* < 0.05, ^*∗∗*^*p* < 0.01, “ns” *p* > 0.05).

Western Blot was used to detect the expression of TLR4, the main receptor of HMGB1, and the key downstream factors NF-*κ*B P65, COX-2, and iNOS, in order to observe the effect of JDNW on the HMGB1-mediated oxidative stress pathway. The results (Figure 9) showed that the expressions of TLR-4, NF-*κ*B P65, COX-2, and iNOS in the ACLF group were significantly increased, the M-5d group being the most obvious. After JDNW treatment for 5, 10, and 15 d, the expression of related signaling pathway proteins was inhibited compared with the model group at the same time point.

## 4. Discussion

The pathological mechanism of ACLF is far from clear, but immune system-related factors are the key cause of ACLF has been widely recognized. ACLF patients have two immune-related pathophysiological characteristics: a state of excessive systemic response and susceptibility to infection [23]. Due to factors such as acute triggers (usually bacterial infections) and pathogens directly or indirectly activate immune cells. In turn, the systemic inflammatory response is aggravated, and a cytokine storm is induced, causing liver cell damage, necrosis, and even extrahepatic tissues. Eventually, liver failure and even the failure of extrahepatic organs are induced [3, 4].

As an important DAMPs factor, extracellular HMGB1 aggravates ACLF progression [24]. Translocation to the cytoplasm is a key process before HMGB1 is released outside the cell membrane. In the current study, we found that in the early injury stage of the ACLF model, the total expression of HMGB1 and the proportion of it in cytoplasm increased significantly and the treatment of JDNW alleviated this increase. A similar increase of HMGB1 is seen in the cytoplasm of L02 cells when D-GaLN damage occurs in vitro. After the intervention of GLY and JDNW serum, the total amount and proportion of HMGB1 in the cytoplasm were significantly reduced. By binding to the TLR4 receptor and increasing transcription activity following release from the cell membrane, HMGB1 regulates the expression of IKB-*α* and NF-kB p65 [25]. As a result, COX-2 and iNOS are activated, and 4-HNE is accumulated, causing lipid peroxidation and oxidative stress [26, 27].

Besides necrosis of hepatocytes plus infiltration of inflammatory cells, excessive apoptosis is another pathological characteristic of ACLF. Studies have confirmed that the intrinsic apoptotic pathway affects the occurrence and development of ACLF [28]. Activating the intrinsic pathway of apoptosis requires mitochondria and is induced by excess ROS and oxidative stress. Bcl-2 counteracts the pro-apoptotic effects of Bax, which digs holes in the mitochondrial outer membrane. Through inhibition of cytochrome c cytoplasmic release, as well as cytochrome c-mediated caspase cascades (caspase-9 is initiated during apoptosis by caspase-3), apoptosis is induced [29]. We previously found that HMGB1 might worsen liver injury in ACLF rats via oxidative stress and then mitochondrial dysfunction, leading to apoptosis [30]. The current study confirmed that extensive apoptosis occurred in ACLF liver tissue, whereas JDNW intervention reduced oxidative stress and apoptosis both in vivo and in vitro. In the ACLF group, the expression of TLR-4, NF-*κ*B p65, COX-2, iNOS, activated caspase-3, and Bax increased, while the anti-mitochondrial pathway apoptosis protein Bcl-2 decreased. The expression of related proteins was reversed after JDNW treatment.

The active components of JDNW may be responsible for this effect through a related mechanism. HPLC was used to analyze the main components of JDNW and their concentrations, including catalpol (CTP; 0.1324 mg/mL), gallic acid (GA; 0.0902 mg/mL), 3,4-dihydroxybenzaldehyde (0.0073 mg/mL), chlorogenic acid CA (0.0266 mg/mL), notoginsenoside R1 (NG-R1; 0.1905 mg/mL), salvianolic acid B (Sal B; 0.5436 mg/mL), and ginsenoside Rb1 (GRb1; 0.1834 mg/mL) [19]. Here, SalB confers protection against hepatic steatosis and inflammation through SIRT1-mediated HMGB1 deacetylation [31]. Sal B significantly ameliorated myocardial I/R injury in a dose-dependent manner, improved cardiac function, attenuated the inflammatory response, and cardiomyocyte apoptosis and expression of the apoptosis proteins Bcl-2 and Bax, as well as HMGB1 and TLR4 [32]. In addition to regulating Caspase-3, Bax, and Bcl-2 expression, Sal B also maintains the permeability of the mitochondrial membrane and ensures basic cellular energy metabolism by regulating their expression [33]. NG-R1 treatment inhibited HMGB1/TLR4/NF-*κ*Bp65, lowered MPO activity, and reduced the severity of acute lung injury [34]. GRb1 inhibits caspase3 activation, downregulates cleaved caspase 3, Bax to reduce hepatocyte apoptosis, and upregulates Bcl-2 protein expression in the liver [35]. By affecting the miR-142/HMGB1/TLR4/NF-*κ*B pathway, CTP protects muscles from SCI by suppressing apoptosis, oxidative stress, and inflammation [36]. HMGB1 induces the production of ECM in human hepatic sinusoids and human umbilical vein endothelial cells when CA is administered [37]. Furthermore, CA activates SIRT1 to inhibit nuclear translocation of HMGB1 and M2 polarization, thereby alleviating *Klebsiella pneumoniae*-induced pneumonia in AMs [38]. It is suggested that the main components of the JDNW formula can inhibit the expression and translocation of HMGB1 and its mediated HMGB1/TLR-4/NF-kB pathway, and have anti-apoptotic and oxidative stress effects. This may partially explain the potential material basis of JDNW ameliorating ACLF liver injury by reducing oxidative stress and apoptosis by inhibiting the HMGB1/TLR-4/NF-*κ*B pathway.

Taken together, in the current study, by revealing that JDNW inhibits the overexpression and translocation of HMGB1, an important DAMP molecular and further affects its mediated oxidative stress injury and apoptosis, it can partially explain the theory of “toxic damage to the liver.” But, the current study still has some limitations. The improvement of oxidative stress and apoptosis by JDNW was demonstrated in this research, as well as its inhibition of Bcl-2 and inhibition of Bax and cleaved caspase-3, indicating the inhibition of apoptosis by JDNW may be associated with the mitochondrial pathway. However, we have not done relevant research on mitochondrial outer membrane permeabilization and mitochondrial functions, which deserves further refinement in future research.

## 5. Conclusion

**Figure 10:**
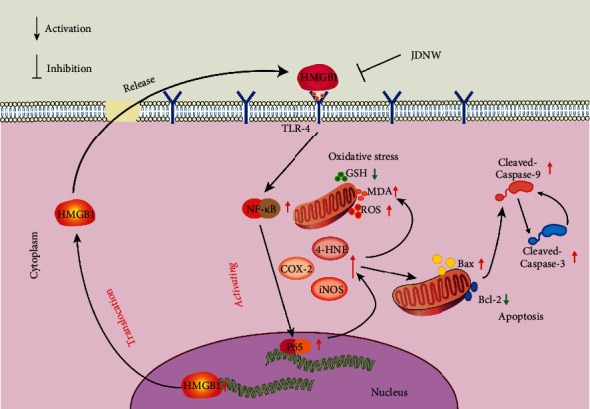
The mechanisms of JDNW inhibit HMGB1 overexpression and translocation, thus the oxidative stress and apoptosis are mediated by the HMGB1/TLR-4/NF-*κ*B pathway in ACLF.

In summary, our current results indicate that JDNW reduced liver injury in ACLF rats, and this effect was a result of its inhibition of the HMGB1/TLR-4/NF-*κ*B pathway, thereby reducing oxidative stress and the occurrence of hepatic apoptosis (Figure 10).

## Data Availability

The data used to support the findings of this study are available from the corresponding author upon reasonable request.

## Supplementary Materials

Supplementary Materials Table 1 Constituents of the JDNW formula. Table 2 Primer sequences for qRT-PCR analyses.
