# Supplementary material for: Jieduan–Niwan Formula Ameliorates Oxidative Stress and Apoptosis in Acute-on-Chronic Liver Failure by Suppressing HMGB1/TLR-4/NF-κB Signaling Pathway: A Study In Vivo and In Vitro
Source: Evid Based Complement Alternat Med. 2022 Jul 15;2022:1833921. doi: 10.1155/2022/1833921 (PMC9307324; doi:10.1155/2022/1833921)
Supplement: Supplementary Materials — Table 1 Constituents of the JDNW formula. Table 2 Primer sequences for qRT-PCR analyses. [file 1833921.f1.zip › Supplementary Material 1.docx]

Supplementary Material

**Suppl. Table 1 Constituents of the JDNW Formula**

| Components | Chinese Name | Dose(g) | Family |
| --- | --- | --- | --- |
| Phyllanthus amarus Schumach. &  Thonn | Ku Wei Ye Xia  Zhu | 30 | Phyllanthaceae |
| Astragalus membranaceus (Fisch.)  Bunge | Huang Qi | 30 | Fabaceae |
| Trichosanthes kirilowii Maxim | Gua Lou | 30 | Cucurbitaceae |
| Lysimachia christinae Hance | Jin Qian Cao | 30 | Primulaceae |
| Viscum coloratum (Kom.) Nakai | Hu Ji Sheng | 30 | Santalaceae |
| Salvia miltiorrhiza Bunge | Dan Shen | 20 | Lamiaceae |
| Rehmannia glutinosa (Gaertn.) DC | Di Huang | 20 | Plantaginaceae |
| Aconitum carmichaeli Debx | Fu Zi | 15 | Ranunculaceae |
| Panax notoginseng (Burkill) F. H. Chen | San Qi | 6 | Araliaceae |
| Curcuma phaeocaulis Valeton | E Zhu | 6 | Zingiberaceae |

**Abbreviation: JDNW formula, Jieduan-Niwan formula**
