# Supplementary material for: Jieduan–Niwan Formula Ameliorates Oxidative Stress and Apoptosis in Acute-on-Chronic Liver Failure by Suppressing HMGB1/TLR-4/NF-κB Signaling Pathway: A Study In Vivo and In Vitro
Source: Evid Based Complement Alternat Med. 2022 Jul 15;2022:1833921. doi: 10.1155/2022/1833921 (PMC9307324; doi:10.1155/2022/1833921)
Supplement: Supplementary Materials — Table 1 Constituents of the JDNW formula. Table 2 Primer sequences for qRT-PCR analyses. [file 1833921.f1.zip › Supplementary Material 2.docx]

Supplementary Material

**Suppl. Table 2 Primer sequences for qRT-PCR analyses**

**Abbreviation: qRT-PCR, Real-time quantitative polymerase chain reaction; Abbreviation: qRT-PCR, Real-time quantitative polymerase chain reaction; HMGB1, high mobility group box 1; GAPDH,glyceraldehyde-3phosphate dehydrogenase.**

| Gene name | Primer sequences (5′ to 3′) |
| --- | --- |
| HMGB1 | Forward 5’-CGAATGTGTCTTTAGCTAGCCCTGT-3’  Reverse 5’-CAGACTGTACCAGGCAAGGTTAGTG-3’ |
| GAPDH | Forward 5’-GGCACAGTCAAGGCTGAGAATG-3’  Reverse 5’-ATGGTGGTGAAGACGCCAGTA-3’ |
